# Micropulse Transscleral Cyclophotocoagulation as Primary Surgical Treatment for Primary Open Angle Glaucoma in Taiwan during the COVID-19 Pandemic

**DOI:** 10.3390/healthcare9111563

**Published:** 2021-11-17

**Authors:** Hsiao-Ling Chang, Shih-Chun Chao, Ming-Tsung Lee, Hung-Yu Lin

**Affiliations:** 1 Department of Ophthalmology, Show Chwan Memorial Hospital, Changhua 500, Taiwan or ado072@show.org.tw (H.-L.C.); arthurking2727@yahoo.com.tw (S.-C.C.); 2 Department of Optometry, Central Taiwan University of Science and Technology, Taichung 406, Taiwan; 3 Department of Optometry, Yuan Pei University, Hsinchu 300, Taiwan; 4 Postdoctoral Fellow National Center for Geriatrics and Welfare Research, National Health Research Institutes, Yunlin 632, Taiwan; lee6717kimo@yahoo.com.tw; 5 Department of Nursing, Hungkuang University, Taichung 433, Taiwan; 6 Department of Optometry, Chung Shan Medical University, Taichung 402, Taiwan; 7 Department of Early Childhood and Family Educare, Chung Chou University of Science and Technology, Changhua 510, Taiwan

**Keywords:** micropulse, transscleral cyclophotocoagulation, diode laser, glaucoma, intraocular pressure

## Abstract

Glaucoma is the leading cause of irreversible blindness worldwide, with primary open angle glaucoma (POAG) accounting for the greatest number of total glaucoma cases. This study aimed to evaluate the efficacy and safety of micropulse transscleral cyclophotocoagulation (MP-TSCPC) as a primary procedure in POAG during the COVID-19 pandemic. We retrospectively analyzed 60 eyes of 52 patients, who were diagnosed with mild-to-end-stage POAG without previous glaucoma surgery and received MP-TSCPC between 1 January 2020 and 31 August 2020. The mean preoperative intraocular pressure (IOP) significantly decreased from 27.8 mm Hg to 19.8, 20.1, 20.3, 20.4, and 20.2 mm Hg at 1, 3, 6, 9, and 12 months, respectively (all *p* < 0.05). The mean number of IOP-lowering medications used significantly decreased from 3.3 at the baseline to 1.6, 1.8, 1.8, 1.9, and 1.9 at 1, 3, 6, 9, and 12 months, respectively (all *p* < 0.001). Total withdrawal of antiglaucoma medications was fulfilled in five patients. The main outcome was achieved in 81.7% at postoperative month 12. The most common adverse effect was transient mydriasis (28.3%). No major complications were encountered. MP-TSCPC seems to be an effective and safe treatment to reduce IOP and the medication burden with minimal vision-threatening complications in mild-to-end-stage POAG patients without previous glaucoma surgery.

## 1. Introduction

Glaucoma is the leading cause of irreversible blindness in the world [1]. Asia alone accounts for more than half of total glaucoma cases worldwide and contains the greatest number of people with primary open angle glaucoma (POAG) and primary angle closure glaucoma (PACG) [2]. Evidence from clinical trials has demonstrated that intraocular pressure (IOP) is strongly correlated with the progression of glaucoma and is the only modifiable risk factor [3,4,5]. Therefore, lowering the IOP plays a crucial role in the management of glaucoma. While medications are generally used as the initial treatment, a large proportion of patients treated with topical medications have concurrent ocular surface disease [6,7]. Besides, topical therapies harbor the predicament of poor adherence, which may contribute to suboptimal IOP control and visual loss [8,9]. Although filtering surgery results in positive outcomes regarding IOP lowering, the risk of long-term complications, including hypotony, infectious endophthalmitis, and bleb-related complications, remains a concern [10,11].

Transscleral cyclophotocoagulation (TSCPC) is a cyclodestructive procedure that utilizes a diode laser near the infrared spectrum at 810 nm, which is strongly absorbed by melanin [12]. TSCPC is introduced to target the pigmented epithelium of the ciliary process, thereby reducing aqueous humor production [13]. Traditionally, TSCPC has been performed using the continuous delivery of laser energy, but the continuous mode has been shown to cause intense collateral tissue damage, resulting in pronounced structural disruption of the ciliary stroma and ciliary muscle [14]. This nonspecific targeting feature of cyclodestruction is thought to contribute to higher rates of vision-threatening complications, including cystoid macular edema, sympathetic ophthalmia, persistent hypotony, and phthisis bulbi, than other glaucoma procedures [15,16]. Consequently, continuous-wave TSCPC (CW-TSCPC) has typically been held out as the last-resort treatment for refractory glaucoma that either has very poor visual potential or is at a high risk for incisional glaucoma surgery [16,17,18,19].

The recently introduced micropulse diode laser has emerged as a potentially effective treatment in various retinal and glaucoma diseases [20,21,22,23,24]. Compared to the continuous mode of traditional diode lasers, micropulse transscleral cyclophotocoagulation (MP-TSCPC) delivers a series of short, repetitive bursts of energy followed by longer rest periods. This technique allows heat to dissipate during the nonoperational cycle of laser conduction. In this way, laser energy delivered in micropulse mode can produce localized coagulative effects in the ciliary body while mitigating overheating and thermal damage to the surrounding tissues [12].

Studies in the literature have demonstrated that MP-TSCPC is an effective method to reduce the IOP and medication burden in various types of glaucoma while exhibiting a more consistent and predictable profile with a lower incidence of adverse effects compared with the traditional continuous mode [25,26]. Nevertheless, most previous studies have evaluated the outcomes of MP-TSCPC in patients with advanced or refractory glaucoma [25,26,27,28,29,30]. On the other hand, the prevailing restriction measures on hospitals during the COVID-19 pandemic and the desire to minimize patient contact during surgery, as well as simplify postoperative care in order to reduce the risk of COVID-19 transmission, had a remarkable impact on the type of surgery offered after the outbreak of COVID-19. A shift in surgical choice in the direction of transscleral diode lasers was documented in the UK after the COVID-19 pandemic [31]. A tendency for less postoperative follow-up, less postoperative interventions, shorter surgical time, improved safety, and anesthetic concerns were the main drive for shifting away from trabeculectomy [31]. In the current study, we aimed to evaluate the efficacy and safety of MP-TSCPC as a primary surgical treatment in mild-to-end-stage POAG in Taiwan during the COVID-19 pandemic. To our knowledge, this is the first study that has investigated the outcomes of MP-TSCPC specifically on mild-to-end-stage POAG patients without prior glaucoma surgery.

## 2. Materials and Methods

This study was conducted in accordance with the tenets of the Declaration of Helsinki and was approved by the Institutional Review Board of the Show-Chwan Memorial Hospital.

### 2.1. Study Design

This is a retrospective chart review of patients who underwent MP-TSCPC at Show-Chwan Memorial Hospital between 1 January 2020 and 31 August 2020.

### 2.2. Participants

Patients offered MP-TSCPC and subsequently included in this study were required to fulfill the following characteristics: (1) mild-to-end-stage POAG and (2) having uncontrolled IOP and/or progression of the disease despite maximally tolerated IOP-lowering medications and were recognized to be poor candidates for further invasive procedures or (3) being intolerant of/nonadherent to the required medical therapy or (4) having deferred incisional glaucoma surgeries due to concerns about the increasing risk of COVID-19 infection during commute, surgery, and attendance in a hospital. The analysis excluded the following: a previous glaucoma surgery, previous cyclodestructive procedure, any intraocular surgery or laser treatment within 3 months before MP-TSCPC, and a history of ocular trauma. All patients had at least 1 year of follow-up after the procedure.

### 2.3. Laser Intervention

The treatment protocol was standardized for all patients, even for retreatment, if necessary. Treatments were performed by an attending glaucoma surgeon (S.C.C.) in the operating room. To carry out the procedure, we used the Cyclo G6 Glaucoma Laser System (Iridex Corporation, Mountain View, CA, USA) with the MicroPulse P3 probe, which emits a diode laser at a wavelength of 810 nm. The device was set to micropulse mode. The laser settings were programmed as follows: laser power 2000 mW, delivered with a duty cycle of 31.33%, equivalent to 0.5 milliseconds of “on time” and 1.1 milliseconds of “off time”, for a total duration of 160 s (80 s per hemisphere). Before the procedure, all patients received a retrobulbar injection of a 1:1 mixture of 2% lidocaine and 2% lignocaine. During the treatment, the MP3 probe was applied perpendicularly to the scleral plane, and the fiberoptic tip of the probe was positioned 3 mm posterior to the limbal margin. The probe was held with a firm and steady pressure over the conjunctiva in a continuous, with slow back-and-forth sliding for four passes (each sweep in one direction took approximately 20 s) over the superior hemisphere, and was then repeated in the inferior hemisphere. The 3 o’clock and 9 o’clock positions were left untreated to avoid damage to the ciliary arteries and nerves. Thinned areas of the sclera were also spared by the laser treatment. At the end of the procedure, an ocular patch was applied. Following MP-TSCPC treatment, all eyes received topical prednisolone acetate 1% (Pred Forte, Allergan Pte Ltd., Singapore) four times daily for a minimum of 1 week, which then could be tapered depending on the grade of inflammation. All preoperative antiglaucoma medications were continued initially and then adjusted at each follow-up visit according to the IOP level. In case a laser-induced IOP-lowering effect was observed, antiglaucoma medications were reduced in a stepwise manner, beginning with oral acetazolamide. Decisions on retreatment or additional incisional surgery were made according to the details of each case and at the clinical discretion of the surgeon.

### 2.4. Examination and Follow-Up

Baseline characteristics regarding age, sex, best-corrected distance visual acuity (BCDVA), preoperative IOP (mm Hg), the number of IOP-lowering medications used, lens status, ocular history (previous surgery and laser therapy), central retinal thickness assessed by optical coherence tomography, and slit-lamp examination findings of the anterior and posterior segments were collected during the month preceding the laser procedure. Pre- and postoperative IOP measurements were obtained with Pascal dynamic contour tonometry (Ziemer Ophthalmic Systems Group Co., Port, Switzerland) [32,33,34,35]. All measurements were taken by an experienced optometrist. BCDVA was evaluated on a Snellen chart and was converted into the logarithm of the minimum angle of resolution (LogMAR). The severity of the glaucoma was assessed according to the Hodapp–Parrish–Anderson staging system on the basis of a visual field examination with a Humphrey^®^ Field Analyzer (Carl Zeiss Meditec Inc., Dublin, CA, USA) preoperatively. This classification system divides mild, moderate, and advanced glaucomatous visual defects [36,37]. Patients unable to perform the Humphrey visual field examination due to severe visual loss were recognized as end-stage glaucoma [38].

After MP-TSCPC treatment, patients were followed up at 1 day, 1 week, 1 month, 3 months, 6 months, 9 months, and 12 months. At each postoperative visit, BCDVA, IOP, and the number of IOP-lowering medications used were recorded. The five classes of IOP-lowering medications used include four topical agents (alpha-adrenergic agonist, beta-blockers, carbonic anhydrase inhibitors, and prostaglandin analog) and one oral agent (acetazolamide). The pain level experienced by patients during and after the procedure was also evaluated. It was graded as mild (tolerable pain without a need for topical analgesia), moderate (tolerable pain with the use of topical analgesia), or severe (intolerable pain even with the use of topical analgesia). Potential complications resulting from MP-TSCPC were recorded, encompassing prolonged anterior chamber reaction persisting for more than 2 weeks, mydriasis, IOP spikes (defined as an increase in the IOP of >25% from the baseline to within 1 month of laser treatment), scleral thinning, cystoid macular edema, hypotony (defined as the IOP lower than 6 mm Hg; mild if IOP was between 6 and 10 mm Hg), and phthisis bulbi.

### 2.5. Outcome Measures

The main outcome measure in this study was the effective lowering of the IOP, defined as an IOP between 6 and 21 mm Hg and/or a 20% reduction from the baseline without an increase in IOP-lowering medications. Retreatment was offered as an option for patients in whom the first session of laser treatment induced insufficient IOP lowering. The secondary outcome measures included the number of IOP-lowering medications used, changes in BCDVA, the number of retreatments, and the incidence of postoperative complications associated with MP-TSCPC.

### 2.6. Statistical Analysis

The statistical analysis was conducted using IBM SPSS Statistics for Windows, version 24.0 (IBM Corp., Armonk, NY, USA). A Student’s *t*-test was used to determine whether the IOP and LogMAR BCDVA at various postoperative time points were significantly different from the baseline. Differences in the number of IOP-lowering medications used between the baseline and various postoperative time points were assessed by the Wilcoxon signed-rank test. The cumulative probability of success after MP-TSCPC treatment was estimated using the Kaplan–Meier curve. Multivariable Cox proportional hazards models were built to identify significant independent predictors of treatment failure by testing several demographic and baseline variables. Their hazard ratios and 95% confidence intervals (CIs) were reported. A *p*-value less than 0.05 was considered statistically significant.

## 3. Results

Fifty-six patients with POAG were treated with MP-TSCPC. Four patients were excluded due to loss to follow-up, resulting in 60 eyes of 52 patients included in our study. The demographic and clinical characteristics of the patients are summarized in Table 1. These patients had a mean age of 65.0 ± 15.8 years. All patients were Han Taiwanese, and 28 patients (53.8%) were male. Most of the patients (80.0%) had moderate glaucoma.

The IOP throughout the study period is presented in Table 2. The mean baseline IOP was 27.8 ± 7.6 mm Hg. The mean IOP was significantly lowered to 20.2 ± 5.9 mm Hg at 1 day (27.3% reduction, *p* < 0.001), to 19.3 ± 6.1 mm Hg at 1 week (30.6% reduction, *p* < 0.001), to 19.8 ± 4.9 mm Hg at 1 month (28.8% reduction, *p* < 0.001), to 20.1 ± 5.8 mm Hg at 3 months (27.7% reduction, *p* < 0.001), to 20.3 ± 6.4 mm Hg at 6 months (27.0% reduction, *p* < 0.001), to 20.4 ± 5.7 at 9 months (26.6% reduction, *p* < 0.001), and to 20.2 ± 4.6 mm Hg at 12 months (27.3% reduction, *p* < 0.001). The greatest magnitude of IOP lowering was noted at 1 week.

The medication burden over time is demonstrated in Table 3. The mean number of IOP-lowering medications used significantly decreased from 3.3 ± 1.3 at the baseline to 1.6 ± 0.7 (51.5% reduction, *p* < 0.001), 1.8 ± 0.9 (45.5% reduction, *p* < 0.001), 1.8 ± 1.1 (45.5% reduction, *p* < 0.001), 1.9 ± 1.3 (42.4% reduction, *p* < 0.001), and 1.9 ± 0.8 (42.4% reduction, *p* < 0.001) at 1, 3, 6, 9, and 12 months, respectively.

The baseline BCDVA in LogMAR ranged from no light perception to 0.0. There was no significant decline in the mean LogMAR BCDVA from the baseline (0.62 ± 0.40) to 1 month (0.59 ± 0.45, *p* = 0.703), 3 months (0.53 ± 0.43, *p* = 0.164), 6 months (0.56 ± 0.49, *p* = 0.369), 9 months (0.59 ± 0.51, *p* = 0.476), or 12 months postoperatively (0.57 ± 0.51, *p* = 0.365), as shown in Table 4.

**Figure 1:**
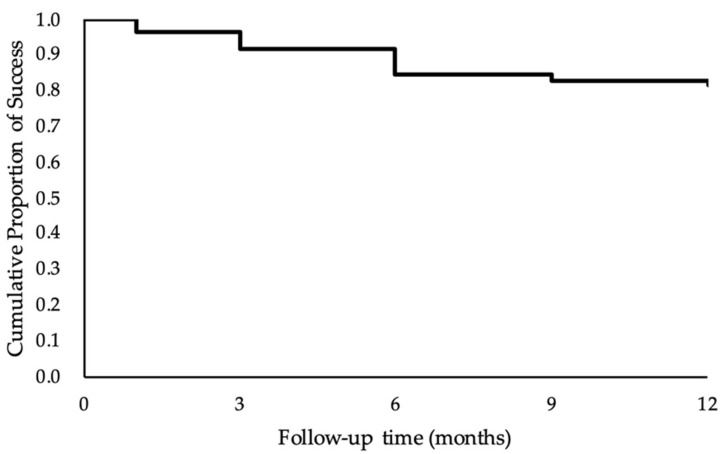
Kaplan–Meier curve representing the probability of success over time after MP-TSCPC treatment. The cumulative probability of success after MP-TSCPC was estimated to be 96.7% at 1 month, 91.7% at 3 months, 85.0% at 6 months, 83.3% at 9 months, and 81.7% at 12 months.

The success rate was 96.7% at 1 month, 91.7% at 3 months, 88.3% at 6 months, 86.7% at 9 months, and 81.7% at 12 months. The Kaplan–Meier curve after MP TSCPC treatment is illustrated in Figure 1. Univariate and multivariate analyses with the Cox proportional hazards model indicated that age, sex, baseline IOP, baseline BCDVA, previous vitrectomy, lens status, IOP at postoperative day 1, and postoperative intraocular inflammation were not significantly associated with treatment failure (all *p* > 0.05), as detailed in Table 5 and Table 6. Retreatment was required in 11 eyes, representing a retreatment rate of 18.3%. Of these 11 eyes, four eyes underwent a second session, and one eye underwent a third session of MP-TSCPC treatment to obtain satisfying IOP lowering during the study period. The mean time between initial treatment and retreatment was 5.0 ± 1.7 months (range 3–7 months). Three of 11 eyes subsequently received trabeculectomy 9 months after MP-TSCPC. These three eyes had end-stage or advanced POAG with higher-than-average IOP at the baseline. No adverse impact of MP-TSCPC on the subsequent trabeculectomy was observed.

The complications following MP-TSCPC during the follow-up period are depicted in Table 7. The most common complication in our series was transient mydriasis (28.3%), followed by inflammatory reactions in the anterior chamber (11.7%), subconjunctival hemorrhage (8.3%), and IOP spikes (6.7%). These complications resolved within one month in all patients. Additionally, cataract progression was observed in 6.1% of eyes that were phakic at the baseline. One patient presented with mild hypotony, but it was transient, being reversed within two weeks upon application of a topical steroid and atropine. Major complications, such as persistent hypotony, sympathetic ophthalmia, or phthisis bulbi, were not encountered during the follow-up period. During the laser procedure, four patients reported experiencing mild pain, whereas another two patients experienced moderate pain, such that additional topical anesthesia was required intraoperatively. Feelings of mild pain in the operated eye or periocular area described by three patients at follow-up examinations only persisted for a maximum of 48 h.

## 4. Discussion

MP-TSCPC has emerged as a promising, noninvasive, and repeatable laser treatment that has been shown to successfully reduce IOP with fewer complications than traditional TSCPC [25]. The reduction in IOP and medication burden in our patients corroborate the increasing number of published reports demonstrating the clinical effectiveness of MP-TSCPC. In this study, IOP significantly decreased from the baseline, with a reduction ranging between 26.6% and 30.6% at different follow-up periods. The IOP-lowering medications used fell by ≥1 medication from the baseline for 49 eyes (81.7%) at 1 month, 47 eyes (78.3%) at 3 months, 44 eyes (73.3%) at 6 months, 43 eyes (71.7%) at 9 months, and 40 eyes (66.7%) at 12 months. We found a significant drop in the number of IOP-lowering medications (1.7 reductions, *p* < 0.001) at the first month, although the number fluctuated over time, as the treatment was adjusted after each visit. In agreement with other studies [27,39,40,41], MP-TSCPC treatment was associated with a marked reduction in the requirements of oral carbonic anhydrase inhibitors. The total withdrawal of acetazolamide was observed in six out of seven patients. All these six patients had a preoperative IOP above 30 mm Hg with oral acetazolamide and four topical IOP-lowering medications used at the baseline. Additionally, five patients (8.3%) were free of all medications from postoperative months 1 (two patients) and months 3 (three patients).

The main outcome was achieved in 81.7% of the eyes after 12 months of follow-up. Further, four eyes (6.7%) and one eye (1.7%) gained satisfying IOP lowering after two and three sessions of MP-TSCPC, respectively. The previously reported rate of treatment success of MP-TSCPC was variable, ranging from 35% to 95.7% over a mean follow-up of 6 to 18 months [25,26,27,28,30,39,42,43,44,45,46,47]. Our rates of success were comparable with other published reports on MP-TSCPC, despite the lower total energy of the laser we employed. A multivariate regression analysis performed by Sarrafpour et al. [40] revealed that a greater reduction in IOP was associated with higher preoperative IOP and a higher strength of laser used intraoperatively (62.5–78.125 J). Al Habash et al. [39], who included cases with predominantly neovascular glaucoma (NVG), attributed their higher success rates and greater IOP reduction to patients’ elevated IOP at the baseline, as well as a higher total laser energy delivered (165 J) than that in other studies. Yelenskiy et al. [44], on the other hand, observed better treatment outcomes in patients with previous filtering surgery or in POAG. We believe that individualized treatment parameters according to patient characteristics and glaucoma subtypes, without going beyond the limits of the safe range [48], may provide more favorable outcomes.

Several studies compared the efficacy and safety of MP-TSCPC in patients who have undergone traditional glaucoma surgery to those without prior glaucoma surgery. However, there have been no consistent results regarding the behavior of MP-TSCPC in subgroup analyses. Magacho et al. [41] revealed that, compared to patients with previous glaucoma surgery, patients receiving MP-TSCPC as the primary procedure required a lower number of laser treatments, with a higher rate of success and preserved visual acuity. Moreover, the rates of complications were lower, with no signs of hypotonia or prolonged inflammation in such patients. This could be explained by the fact that these patients had less advanced glaucoma. Another justifiable explanation is that the eyes of these patients were in better condition for laser treatment, and as a consequence, their outcomes were more favorable.

The IOP-lowering effect achieved with MP-TSCPC in our patients was seen as early as one day post-MP-TSCPC, which was consistent with previous studies demonstrating the rapidity of IOP reduction [44,46]. Tan et al. [46] assumed that the rapid and sustained reduction in IOP may be mainly attributed to the enhancement of uveoscleral outflow. Decreased aqueous production caused by inflammation should result in only a transient reduction in IOP that vanishes as the inflammation settles. Two experimental studies, performed on postmortem human eyes and porcine eyes by Schubert et al. [49] and performed on monkey eyes by Liu et al. [50], both identified an increased uveoscleral outflow after transscleral laser treatment targeted over the pars plana.

Eyes that underwent retreatment with MP-TSCPC or received subsequent trabeculectomy appeared to be less responsive to MP-TSCPC, presumably due to higher IOP than the mean value and poorer visual potential preoperatively, although the baseline IOP and visual acuity were not recognized as significant predictors for treatment failure in the present study. In a retrospective multicenter study, Yelenskiy et al. [44] observed that the baseline IOP in the retreatment subgroup was significantly higher than that in eyes not requiring retreatment and was the only independent predictor of repeat MPTSCPC. Aquino et al. [21] reported the highest retreatment rate of 46% among previous publications, which may be explained by a higher baseline IOP, a greater proportion of NVG patients, and a lower level of the total energy use (62.6 J) compared to our study (100 J). Additionally, Tekeli et al. [51] compared the efficacy of two different duration protocols (160 s vs 240 s) and found that the only variable factor associated with a higher hazard for retreatment was the treatment duration. A higher treatment duration (240-s group) tends to be more effective for adequate and sustained IOP control. On the contrary, Vig et al. [43] reported the effectiveness of a reduced energy protocol (90 s of laser with settings of 2000 mW/cm^2^); however, all subjects in their study had either undergone intraocular surgery (58.6% filtration surgery) or used a continuous-wave diode laser prior to micropulse treatment, and their study was limited by a small sample size (*n* = 29), as well as short duration of follow-up (6 months). On the other hand, the success rate in our study appeared to decrease over time, which was also described in previous studies [28,52]. A study including all POAG eyes by Tong et al. [52] found that the IOP-lowering effect of MP-TSCPC treatment was not permanent, and definitive glaucoma surgery was needed in a number of patients. Reasons for the decline of the success rate over time have not been well-identified. Yamashita et al. [53] evaluated the regeneration of ciliary epithelium after cryo injury. They provided evidence of the regenerative ability of the pigmented and nonpigmented epithelial cell layers, and the recovery of the IOP slightly lagged behind the regeneration of the nonpigmented epithelium. Further prospective studies are necessary to investigate the possible mechanisms associated with IOP recovery after MP-TSCPC.

Although better outcomes with regards to efficacy are demonstrated in studies using higher amounts of energy in the treatment, the rates of complications are also higher [41,54]. Transient mydriasis was the most common complication in our study, resulting in visual complaints such as blurry vision or glare in some patients. This observation eventually resolved within one month. Postoperative reversible mydriasis has been described in several studies [39,41,55,56,57]. Dorairaj et al. [56] identified that myopic females with brown irises were more likely to develop this complication. Radhakrishnan et al. [57] reported higher odds of persistent mydriasis in those of Asian descent (odds ratio = 13.07, *p* < 0.001) and in phakic eyes (odds ratio = 3.12, *p* = 0.014). Causes of this condition may be multifactorial, while the most widely accepted mechanisms are the occlusion of iris vessels and iris ischemia secondary to an acute elevation in IOP leading to ischemic atrophy of the iris sphincter, with consequent pupil dilation [56].

Apprehension of visual loss is one of the factors that has conventionally limited the use of traditional TSCPC as the last-resort treatment for patients with refractory end-stage glaucoma. One eye of a patient having improper glycemic control and proliferative diabetic retinopathy with diabetic macular edema at the baseline was found to have lost ≥2 lines of LogMAR visual acuity at postoperative month 6. This patient had suspended scheduled monthly intravitreal injection of anti-vascular endothelial growth factor since three months before MP-TSCPC. This visual loss was considered more presumably to be related to an underlying diabetic retinopathy and the nature of the retinopathy worsening. In a study by Varikuti et al. [42], who enrolled only patients with good central vision of ≥20/60, deterioration in visual acuity was observed in 20.83% of patients after 12 months of follow-up. A high rate of cataract progression after MP-TSCPC was suggested to account for most of the visual decline. In our study, cataract progression was found in three eyes (6.1%) at postoperative month 3. It remains inconclusive whether the cataract progression occurred due to intraocular inflammation resulting from the MP-TSCPC or whether these cataracts would have progressed regardless of the MP-TSCPC procedure. The results of our study support the favorable safety profile that has been demonstrated for MP-TSCPC [40,47,58]. Conversely, a broader range of complications and higher rates of prolonged intraocular inflammation were shown by the publications of Williams et al. [30] and Emanuel et al. [54]. A possible cause could be the longer treatment times—300 s and 319 s, respectively—used in their studies. A second reason might be found in the characteristics of the study populations. Those authors included 30.4% and 29% African-Americans, respectively. Williams et al. [30] found that non White descents were significantly more prone to developing prolonged inflammation after MP-TSCPC (odds ratio = 3.61; 95% confidence interval = 1.27–10.23; *p* = 0.02). The absence of prolonged inflammation and other major complications is noteworthy in our entirely Han Taiwanese series. This result could be attributed to the shorter treatment time of our laser protocol, the mostly moderate glaucoma, and the surgery-naive history of our patients, as well as the better ocular conditions at the baseline for treatment in our patients.

The COVID-19 pandemic has placed an unprecedented impact on the healthcare system globally, hindering the continuous care of patients with chronic diseases, affecting the health-seeking behavior of patients, and influencing the clinical discretion of surgeons. Subathra et al. [59] described as much as 88% of follow-up visits of glaucoma patients affected in a tertiary center in South India, and 57.3% of the patients were nonadherent to glaucoma medication during the pandemic lockdown in their study. The main impediments encountered during the pandemic for medication adherence were difficulty in the accessibility of medication (54.81%), financial difficulties (30.29%), and the fact that patients did not feel much improvement with the eye drops (20.19%). On the other hand, the choices of anesthesia and surgery were significantly influenced by the restriction measures and the concerns about the risk of exposure for healthcare providers. To operate under local anesthesia with day care and avoid general anesthesia was preferred when possible [31,60,61]. Less invasive glaucoma procedures requiring fewer postoperative visits and fewer postsurgical interventions were favored at the discretion of the operating surgeon [60,62]. A study examining the effects of the COVID-19 pandemic on glaucoma surgical practices within the UK demonstrated 43 respondents (61%) reported modifying their glaucoma surgery practice subsequent to the onset of the COVID-19 pandemic. The number of trabeculectomy, glaucoma drainage devices, and minimally invasive glaucoma surgery performed was reduced. Instead, a diode laser (both micropulse and conventional transscleral cyclodiode) was the most common alternative procedure. Additionally, Rajendrababu et al. [63] compared the glaucoma procedures performed before and during the COVID-19 pandemic (23 March 2020–23 June 2020 vs. 23 March 2019–23 June 2019). They found an increase in the proportions of transscleral diode cyclophotocoagulation (45/115 (39.13%) vs. 133/939 (14.16%); *p* = 0.0001) and a decrease in the proportions of incisional glaucoma surgeries (70/115 (60.86%) vs. 806/939 (85.83%); *p* < 0.001) during the pandemic. Taiwan, like other nations across the world, implemented mass quarantines, restrictions of public transport, strict social distancing protocols, the closure of entertainment and sports venues, and deferral of elective surgery and routine health appointments. We were confronted with the dilemma of endeavoring to minimize irreversible visual loss due to the progression of diseases, as well as postponed treatment, while being attentive that the process of hospital visits increasing the risk of viral transmission for the patients and other close contacts. Many glaucoma surgical interventions have been suspended in mild-to-moderate cases where there is no evidence of progression or no urgent visual threat. Nevertheless, the termination of the pandemic is uncertain and whether these patients are safe for deferral or not. Since there has been little specific guidance for glaucoma surgery in the COVID-19 era, our study suggesting promising outcomes of MP-TSCPC in mild-to-moderate POAG may support and accelerate the trend away from incisional surgery towards less invasive procedures, such as MP-TSCPC treatment.

Our study has several limitations. Since the study was retrospective in nature, there was no randomization to any number of treatment options. Additionally, it remains to be determined what factors are predictors of treatment failure with MP-TSCPC. Finally, this study was limited to a small sample size, with a relatively brief follow-up period, and the absence of a comparative group receiving alternative treatment, such as incisional surgery. Nevertheless, it is important to recognize that our study aimed to evaluate the outcomes of a previously validated approach offered as the primary procedure in the management of POAG during the period of the COVID-19 outbreak.

## 5. Conclusions

In conclusion, MP-TSCPC using our standardized protocol appears to be an effective primary surgical treatment for reducing the IOP and medication burden with minimal vision-threatening complications in patients with mild-to-end-stage POAG. A prominent reduction in IOP can be expected within 1 week, although some patients may need retreatment to obtain a sustained IOP-lowering effect. Further prospective, multicenter studies may be necessary to analyze the long-term effectiveness and potential late complications of MP-TSCPC, as well as to better determine if MP-TSCPC is worth considering as a viable alternative in the earlier course of glaucoma management.

## Figures and Tables

**Table 1:** Patient demographic and clinical characteristics.

Characteristic	N ± SD (%)
Age (years)	65.0 ± 15.8 (range 19–89)
Sex	
Men	28 (53.8%)
Women	24 (46.2%)
Ethnicity	
Han Taiwanese	52 (100%)
Laterality	
Right	25 (48.1%)
Left	19 (36.5%)
Bilateral	8 (15.4%)
Glaucoma severity ^†^	
Mild	5 (8.3%)
Moderate	48 (80.0%)
Advanced	6 (10.0%)
End-stage ^‡^	1 (1.7%)
Lens status	
Phakic	49 (81.7%)
Pseudophakic	11 (18.3%)
Previous ocular surgery or laser treatment	
Pars plana vitrectomy	5 (8.3%)
Intravitreal injection	4 (6.7%)
Radial keratotomy	2 (3.3%)
Laser in situ Keratomileusis	4 (6.7%)
Patients treated with oral carbonic anhydrase inhibitor	7 (13.5%)

Abbreviations: N, number; SD, standard deviation. † Glaucoma severity was assessed according to the Hodapp–Parrish–Anderson staging system. This system divides mild, moderate, and advanced glaucomatous visual defects. ‡ The end-stage glaucoma was defined by visual acuity <20/200 or unavailable to perform the Humphrey visual field examination attributable to glaucoma.

**Table 2:** Intraocular pressure at various postoperative time points compared to the baseline.

	Intraocular Pressure (mm Hg)		
Time	Mean	SD	*p*-Value
Baseline	27.8	7.6	
1 day	20.2	5.9	<0.001
1 week	19.3	6.1	<0.001
1 month	19.8	4.9	<0.001
3 months	20.1	5.8	<0.001
6 months	20.3	6.4	<0.001
9 months	20.4	5.7	<0.001
12 months	20.2	4.6	<0.001

Abbreviation: SD, standard deviation. *p*-values denote paired *t*-test results compared to the baseline. Significant at *p* <  0.05.

**Table 3:** The number of IOP-lowering medications used at various postoperative time points compared to the baseline.

	Number of IOP-Lowering Medications ^†^			
Time	Mean ± SD	Median ± IQR	Range	*p*-Value
Baseline	3.3 ± 1.3	3 ± 2	1–5	
1 month	1.6 ± 0.7	1 ± 1	0–2	<0.001
3 months	1.8 ± 0.9	1 ± 1.5	0–2	<0.001
6 months	1.8 ± 1.1	2 ± 1	0–3	<0.001
9 months	1.9 ± 1.3	2 ± 1	0–3	<0.001
12 months	1.9 ± 0.8	2 ± 1.5	0–3	<0.001

Abbreviations: IOP, intraocular pressure; SD, standard deviation; IQR: interquartile range. † Five classes of IOP-lowering medications used included four topical agents (alpha-adrenergic agonist, beta-blockers, carbonic anhydrase inhibitors, and prostaglandin analogue) and one oral agent (acetazolamide). *p*-values denote Wilcoxon signed-rank test results compared to the baseline. Significant at *p* < 0.05.

**Table 4:** BCDVA at various postoperative time points compared to the baseline.

	LogMAR BCDVA		
Time	Mean	SD	*p*-Value
Baseline	0.62	0.40	
1 month	0.59	0.45	0.703
3 months	0.53	0.43	0.164
6 months	0.56	0.49	0.369
9 months	0.59	0.51	0.476
12 months	0.57	0.51	0.365

Abbreviations: SD, standard deviation; BCDVA, best-corrected distance visual acuity. *p*-values denote paired *t*-test results compared to the baseline. Significant at *p* < 0.05.

**Table 5:** Univariate Cox proportional hazards models to investigate the significant independent predictors of treatment failure.

Variables	aHR	95% CI	*p*-Value
Age			
≥60	Reference		
<60	1.32	0.56–3.08	0.529
Sex			
Female	Reference		
Male	0.88	0.37–2.10	0.773
Baseline IOP			
≥30	Reference		
<30	0.95	0.40–2.26	0.903
Baseline LogMAR BCDVA			
≥0.60	Reference		
<0.60	0.93	0.35–2.01	0.816
Previous vitrectomy			
No	Reference		
Yes	0.91	0.33–2.46	0.849
Lens status			
Pseudophakia	Reference		
Phakia	0.76	0.29–1.94	0.565
IOP at postoperative day 1			
≤21	Reference		
>21	1.68	0.93–1.77	0.091
Postoperative intraocular inflammation			
<2 weeks	Reference		
≥2 weeks	1.47	0.76–2.36	0.369

Abbreviations: aHR, adjusted hazard ratio; CI, confidence interval; IOP, intraocular pressure. *p*-values were determined by means of the Cox proportional hazards model. Significant at *p* < 0.05.

**Table 6:** Multivariate Cox proportional hazards models to investigate the significant independent predictors of treatment failure.

Variables	aHR	95% CI	*p*-Value
Age			
≥60	Reference		
<60	1.30	0.47–3.63	0.617
Sex			
Female	Reference		
Male	0.68	0.25–1.87	0.451
Baseline IOP			
≥30	Reference		
<30	0.93	0.39–2.24	0.871
Baseline LogMAR BCDVA			
≥0.60	Reference		
<0.60	0.92	0.37–2.18	0.843
Previous vitrectomy			
No	Reference		
Yes	0.81	0.26–2.54	0.717
Lens status			
Pseudophakia	Reference		
Phakia	0.78	0.26–2.33	0.652
IOP at postoperative day 1			
≤21	Reference		
>21	1.51	0.89–1.82	0.104
Postoperative intraocular inflammation			
<2 weeks	Reference		
≥2 weeks	1.20	0.61–2.96	0.583

Abbreviations: aHR, adjusted hazard ratio; CI, confidence interval; IOP, intraocular pressure. *p*-values were determined by means of the Cox proportional hazards model. Significant at *p* < 0.05.

**Table 7:** Summary of the complications following MP-TSCPC during the follow-up period.

Complications	N (%)
Transient mydriasis	17 (28.3%)
Inflammatory reaction in the anterior chamber	7 (11.7%)
Subconjunctival hemorrhage	5 (8.3%)
IOP spikes	4 (6.7%)
Cataract progression	3 (6.1%)
Vision loss ≥ 2 lines	1 (1.7%)
Mild hypotony	1 (1.7%)
Pain during the procedure	
Mild	4 (7.7%)
Moderate	2 (3.8%)
Pain during the early postoperative period	
Mild	3 (5.8%)

Abbreviations: MP-TSCPC, micropulse transscleral cyclophotocoagulation; N, number; IOP, intraocular pressure.

## Data Availability

The datasets generated during and/or analyzed during the current study are not publicly available but are available from the corresponding author upon reasonable request.
